# *Toxoplasma gondii* and a Cancer Biology Dichotomy: A Systematic Review of Experimental Studies of Its Antitumor and Pro-Tumor Effects

**DOI:** 10.3390/pathogens15040351

**Published:** 2026-03-26

**Authors:** Saachi Jhandi, Brenda Anissa Vera, Julian Galindo, Jose G. Montoya, Despina G. Contopoulos-Ioannidis

**Affiliations:** 1 Spencer Fox Eccles School of Medicine, University of Utah, Salt Lake City, UT 84112, USA; saachi.jhandi@hsc.utah.edu; 2 David Geffen School of Medicine, University of California Los Angeles, Los Angeles, CA 90095, USA; bvera@mednet.ucla.edu; 3 Dr. Jack S. Remington Laboratory for Specialty Diagnostics, Palo Alto Medical Foundation, Sutter Health, Palo Alto, CA 94301, USA; ja22galindo@hotmail.com (J.G.); jose.montoya2@sutterhealth.org (J.G.M.)

**Keywords:** *Toxoplasma gondii*, *T. gondii*, acute infection, chronic infection, recombinant antigens, parasite antigens, cancer biology, systematic review, experimental studies, in vitro, in vivo, antitumor, pro-tumor, protumor, translational research, cancer immunotherapy

## Abstract

*Toxoplasma gondii* (*T. gondii*) is an intracellular parasite known to modulate host immunity and cellular signaling, raising interest in its potential influence on cancer biology. A systematic review was conducted to evaluate experimental evidence on the antitumor or pro-tumor effects of *T. gondii* infection and parasite-derived antigens and to categorize the underlying mechanisms. PubMed was searched through 9 September 2024, and 54 eligible experimental studies were included (41 in vivo, 10 in vitro, and three combined). Forty-six studies reported antitumor effects, two pro-tumor effects, one stage-dependent divergent effects (acute infection/antitumor vs. chronic infection/pro-tumor), and five highlighted *T. gondii*-associated cancer-pertinent signaling pathways. Antitumor effects were observed following acute infection and exposure to parasite antigens, certain recombinant proteins, and exosomal microRNA miR-155-5p. Dominant mechanistic categories included activation of innate and adaptive immunity and reversal of tumor microenvironment immunosuppression (notably Th1-driven IL-12/IFN-γ responses, antitumor M1 macrophage polarization), induction of apoptosis, anti-angiogenesis, molecular mimicry and modulation of cancer-pertinent pathways. Conversely, pro-tumor effects were seen with chronic infection and exposure to ROP18 effector protein and miR-21. Future translational research should focus on rigorous evaluation of the safety and efficacy of attenuated non-replicating *T. gondii* strains and/or select recombinant antigens for potential cancer *T. gondii*-based immunotherapy.

## 1. Introduction

*Toxoplasma gondii* (*T. gondii*) is a globally prevalent intracellular protozoan parasite. Following acute infection, the organism transitions into a lifelong chronic latent stage through formation of tissue cysts. *T. gondii* has profound immunomodulatory capacity, capable of reprogramming host immune and signaling pathways at both cellular and molecular levels. Over the past two decades, an apparent paradox has emerged: acute infection with *T. gondii* exhibits potent antitumor effects, whereas chronic infection is associated with tumor-promoting consequences in experimental models.

The antitumor potential of *T. gondii* was first recognized in murine models in the early 1970s at the Remington JS laboratory, which showed that sulfadiazine-treated acute *T. gondii* infection was associated with suppressed tumor growth and prolonged survival [1,2]. In subsequent experimental studies, the antitumor effects of acute *T. gondii* infections were attributed to strong induction of pro-inflammatory T helper 1 (Th1) immunity [3], driven by interleukin-12 (IL-12) and interferon-γ (IFN-γ), which enhanced cytotoxic T cell and natural killer (NK) cell activity [4]. *T. gondii* was also shown to secrete an array of effector proteins—including rhoptry and dense-granule proteins—that directly manipulated host signaling pathways. For example, the *Toxoplasma gondii* rhoptry kinase ROP16, a secreted effector protein released from parasite invasion organelles called rhoptries, and the dense granule protein GRA16, an effector secreted from post-invasion dense granules, modulate host p53 and phosphatase and tensin homolog (PTEN) signaling pathways, inducing apoptosis and cell-cycle arrest [5,6,7,8]. Moreover, GRA6 and GRA17 enhanced antitumor immune responses and sensitized tumors to immunotherapy [9,10]. These findings position *T. gondii* and its parasite-derived antigens as promising platforms for cancer immunotherapy.

In contrast, chronic *T. gondii* infection was shown to be associated with persistent low-grade inflammation, and transcriptomic reprogramming that favored tumor growth [11]. Moreover, experimental studies have shown that *T. gondii* released exosomal microRNA miR-21 inhibited tumor-suppressor genes and promoted glioma proliferation [12] and rhoptry protein ROP18 inhibited mitochondrial apoptosis and enhanced tumor survival [13]. These findings indicate that *T. gondii* exerts opposing biological effects depending on whether infection is acute or chronic and according to the type of *T. gondii* antigen exposure thereof. Understanding the molecular determinants of this divergence is critical in the potential development of safe *T. gondii*-based immunotherapeutic strategies. Furthermore, sero-epidemiological studies in humans also reveal a higher incidence of *T. gondii* seropositivity among patients with diverse cancers, particularly brain tumors, but causal association could not be determined by such studies [14,15].

A prior review had explored experimental studies of the antitumor and pro-tumor effects of *T. gondii* [16], but since then, numerous additional mechanistic experimental studies have emerged—for diverse tumor models and parasite preparations thereof—exploring both the antitumor and pro-tumor effects of *T. gondii.* An updated systematic review and synthesis of the experimental evidence was therefore warranted to further clarify and unify the mechanisms by which *T. gondii* influences tumor biology and to provide an updated guidance for future translational research with potential implications for cancer immunotherapy.

This systematic review aimed to: (1) identify experimental in vitro and in vivo studies explicitly evaluating the antitumor and pro-tumor effects of *T. gondii* (and mechanisms thereof), and consider also studies with cancer-pertinent signaling pathway analyses post-*T. gondii* infections; (2) classify cancer effects by type of experimental model (in vivo or in vitro) and type of *T. gondii* exposure (infection stage [acute vs. chronic] and parasite preparation thereof [live wild-type vs. live attenuated strain vs. parasite lysate antigens or recombinant antigens]); (3) identify dominant mechanistic categories (and mechanistic details thereof) explaining the antitumor and pro-tumor effects of *T. gondii* across different experimental models; and (4) determine whether the cumulative evidence supports a coherent biological framework for the role of *T. gondii* in tumor biology that could lead to clinical applications in cancer immunotherapy.

By delineating these mechanistic patterns, this systematic review provides an up-to date evidence-based framework to guide future translational efforts aimed at safely exploiting the parasite–host immune interactions for cancer immunotherapy.

## 2. Materials and Methods

### 2.1. Eligibility Criteria

This systematic review searched for in vitro and in vivo experimental studies that explicitly explored the role of *T. gondii* in tumor biology for its antitumor or pro-tumor effects. A comprehensive search of PubMed was performed; PubMed was last searched 9 September 2024. No date or language filters were applied; however, at the eligibility screening stage, only studies published in English or Spanish were considered to ensure accurate data extraction by our team of investigators. We used different search strategies with different combinations of parasite terms and cancer terms, as shown in Appendix A. Moreover, given the particular interest in the scientific literature of the association between chronic *T. gondii* infections and brain tumors, additional search terms were used for different types of brain tumors to increase the sensitivity of the search strategy for central nervous system (CNS)-related tumor models. Experimental studies were considered eligible for inclusion if they had a clear focus on the mechanistic relationship between *T. gondii* and cancer, exploring its antitumor or pro-tumor effects.

To improve completeness, the reference lists from key prior reviews were also hand-searched for potential additional eligible studies.

Reviews, editorials, commentaries, clinical case reports and case series, epidemiologic association studies and seroprevalence studies (exploring only the association between *T. gondii* seropositivity and cancer, without experimental mechanistic insight) were excluded.

### 2.2. Study Selection

**Figure 1:**
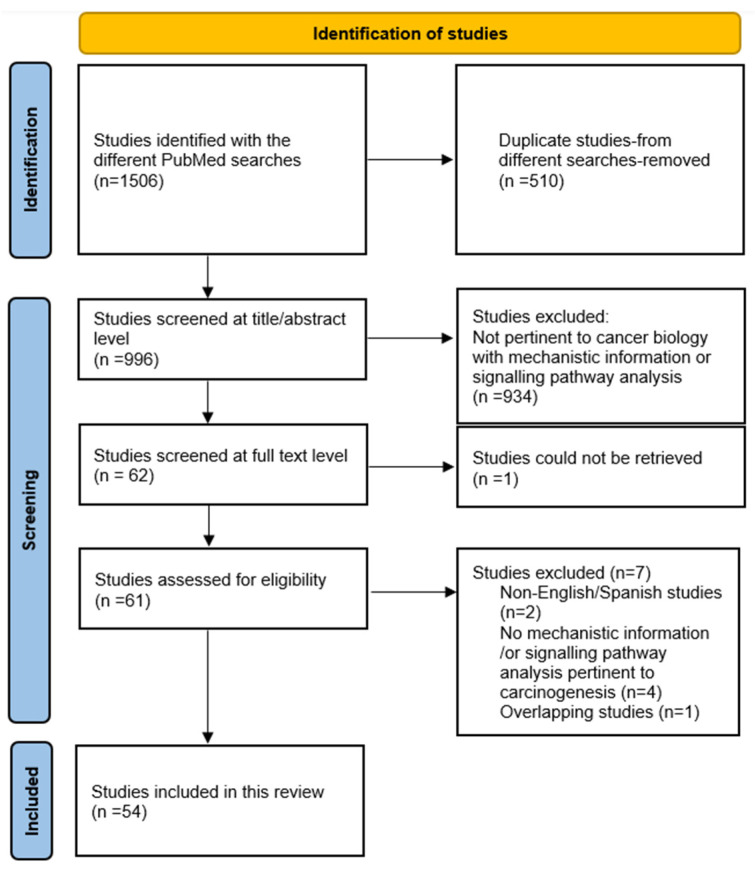
Identification of 54 eligible experimental studies.

Initial screening for potentially eligible studies was done at the title/abstract level (D.G.C.-I.). Subsequently, all potentially eligible studies were screened at the abstract and full-text level (S.J., J.G.). Inclusion decisions requiring additional arbitration were resolved through consensus with D.G.C.-I.; final adjudication of the list of the included articles was done by D.G.C.-I. The flow chart for the identification of eligible studies is shown in Figure 1.

### 2.3. Data Extraction

All eligible articles were reviewed at the full-text level. A shared folder with all eligible studies was created and used by all study authors. The following data were manually extracted from all eligible studies: publication details (author, journal, year), experimental model (in vitro or in vivo), tumor type (cancer cell line or animal model), type of *T. gondii* exposure (infection with *T. gondii* strain [live wild-type, live attenuated mutant, irradiated or autoclaved stain] or exposure to antigen preparations [formalin-fixed tachyzoites, toxoplasma lysate antigens, or recombinant proteins], or exposure to exosomes), infection stage model (acute or chronic), direction of cancer effect (antitumor or pro-tumor), identified/proposed dominant mechanistic category(ies) for antitumor or pro-tumor effect (and mechanistic pathway details thereof), signaling pathway/transcriptomic analyses pertinent to cancer and author-proposed translational implications. A summary vignette was also created for each study describing the mechanistic details of the proposed antitumor or pro-tumor effects.

The identified antitumor and pro-tumor effects were categorized under eight dominant mechanistic categories defined a priori: (a) antitumor effect via innate and adaptive immune activation and reversal of tumor microenvironment (TME) immunosuppression; (b) antitumor effect via apoptosis induction; (c) antitumor effect via anti-angiogenesis; (d) antitumor effect via molecular mimicry; (e) antitumor effect via cell-intrinsic pathways; (f) pro-tumor effect; (g) dual/stage-dependent divergence (acute infection/antitumor effect vs. chronic infection/pro-tumor effect); and (h) other (e.g., signaling pathway/transcriptomic analyses of cancer-pertinent pathways post-*T. gondii* experimental infections (with or without a specific tumor experimental model).

It is acknowledged that there is overlap between the above mechanistic categories and some studies could have been categorized under more than one mechanistic category; however, for the purpose of this systematic review, each study was categorized under the dominant mechanistic category described in the study.

All data extraction was done in duplicate. Initial data extraction was done by S.J. and J.G.; data curation/complementation and clarifications about the extracted mechanistic categories and classification thereof were done by additional independent reviewers (S.J., B.A.V., D.G.C.-I.). Discrepancies were solved by consensus and final arbitration was done by D.G.C.-I.

### 2.4. Data Synthesis

Because of the large heterogeneity in experimental models and outcome measures, quantitative meta-analysis of the proposed cancer-associated effects of *T. gondii* was not feasible and results were summarized only descriptively. Aggregated results are presented in Table 1 (study characteristics) and Table 2 (mechanistic classification).

A compilation of vignettes was also created (for each study), with more mechanistic details for the antitumor or pro-tumor effects, and is available in Supplemental Material Table S1.

Risk-of-bias tools, such as the SYRCLE [58] for interventional animal studies, were not applicable for this systematic review as most of the included studies were either in vitro-only studies or a combination of in vitro and in vivo studies, and their main focus was exploration of mechanistic pathways pertinent to cancer—including signaling pathway analyses—rather than evaluation of efficacy, precluding the meaningful implementation of such a tool.

### 2.5. Reporting, Registration, Ethics

This systematic review followed the Preferred Reporting Items for Systematic Reviews and Meta-Analyses (PRISMA) 2020 guidelines [59] and was registered in the Open Science Framework (OSF) Registry of the Center for Open Science [60]. Moreover, because the study synthesized published data and did not involve registration of any human or animal subjects, institutional ethics approval was not required.

## 3. Results

### 3.1. Study Characteristics

Fifty-four eligible experimental studies, published between 1971 and 2024, were identified. The flow chart for the identification of the eligible experimental studies is shown in Figure 1. Forty-one studies used in vivo models only, 10 in vitro models only and three both in vivo/in vitro models (Table 1). In total, 17 diverse tumor models were used in in vitro or in vivo experimental studies, including solid and non-solid tumors, such as breast cancer, colorectal cancer, Ehrlich ascites/solid cancer, glioma/glioblastoma, hepatocarcinoma, leukemia/lymphoma, lung cancer/non-small cell lung cancer, melanoma, neuroblastoma, ovarian cancer, pancreatic cancer, and sarcoma/fibrosarcoma models. The experimental interventions included *T. gondii* infections with wild-type or attenuated mutants (e.g., Δcps, Δldh, Δompdc, Δgra5, Δgra17), inactivated or autoclaved *T. gondii* strains (autoclaved *T. gondii* vaccine-ATV), exposure to parasite antigens (e.g., tachyzoite lysate antigen (TLA)), dendritic cell (DC)-derived exosomes, or recombinant parasite effector proteins (ROP16, ROP18, GRA5, GRA6Nt, GRA8, GRA15II, GRA16, GRA17) (Table 1). Forty-six studies reported antitumor effects, two reported pro-tumor effects, one reported stage-dependent divergent effects (acute infection/antitumor effect vs. chronic infection/pro-tumor effect), and five identified signaling pathways pertinent to cancer after *T. gondii* infections using transcriptomic analyses (in experimental non-tumor and/or tumor models).

**Figure 2:**
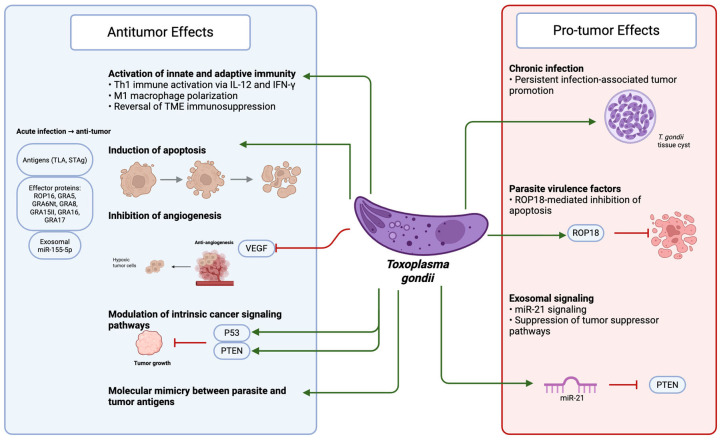
*Toxoplasma gondii* modulation of tumor biology. Footnote: Shown are examples of main mechanistic categories; for detailed descriptions, please see Table 1. (Figure 2 was created in BioRender via use of academic institutional license (Jhandi, S. (2026)), https://BioRender.com/u0ptnne, accessed on 12 March 2026). Abbreviations: DC-: dendritic cell; GRA-: dense granule protein; IL-: interleukin; INF-: interferon; miR: micro RNA; PTEN: phosphatase and tensin homolog; ROP-: rhoptry protein; STAg: soluble tachyzoite antigens; *T. gondii: Toxoplasma gondii*; Th1-: T helper cell type 1-; TLA: tachyzoite lysate antigens; VEGF: vascular endothelial growth factor.

The identified cancer mechanistic pathways were categorized into eight predefined broader mechanistic categories that were decided a priori (Table 2). These broader mechanistic categories (and examples of identified specific mechanistic patterns thereof) included: (a) antitumor effect via innate and adaptive immune activation and reversal of tumor microenvironment (TME) immunosuppression (e.g., Th1 immune activation via IL-12/IFN-γ signaling pathways, increase in CD4^+^/CD8^+^ T cell infiltration into the tumor microenvironment (TME), transformation of “cold” tumors to “hot” tumors, macrophage and NK cell activation, dendritic cell (DC) priming (DC-derived exosomal microRNA [miR155-5p]), macrophage polarization to antitumor M1 macrophage phenotype, decrease in myeloid-derived suppressor cells (MDSC) and regulatory T cells (Treg)); (b) antitumor effect via apoptosis induction (e.g., mitochondria “metabolic resuscitation”, caspase activation); (c) antitumor effect via anti-angiogenesis (e.g., VEGF suppression and reduced tumor vascularization); (d) antitumor effect via molecular mimicry (e.g., cross-reactivity between *Toxoplasma* antigens and tumor antigens); (e) antitumor effect via cell-intrinsic pathways (e.g., modulation of PTEN/p53/p21 tumor suppressive signaling pathways, inhibition of AKT, STAT3, and NF-κB pathways, telomerase suppression, and regulation of tumor-suppressive microRNA); (f) pro-tumor mechanisms (e.g., oncogenic microRNA induction, inhibition of tumor suppressor genes, and inhibition of mitochondrial apoptosis); (g) dual stage-dependent divergent effects (acute infection/antitumor effect vs. chronic infection/pro-tumor effect); and (h) signaling pathway/transcriptomic analysis after *T. gondii* infection pertinent to cancer. Mechanistic details about the antitumor or pro-tumor effects of *T. gondii* are listed in Supplementary Table S1. Figure 2 summarizes graphically examples of the main mechanistic categories.

### 3.2. Antitumor Effect via Innate and Adaptive Immune Activation/Reversal of Tumor Microenvironment Immunosuppression

This was the most commonly described mechanistic category in the included studies (28 studies [52%]) (Table 2). Sulfadiazine-treated acute *T. gondii* infections reduced tumor growth by activating microglia and macrophages and by leading to a macrophage-driven non-specific cytotoxicity via a non-phagocytic mechanism [1,2]. Acute infection restored NK cytotoxicity in NK-deficient mice through IFN-dependent signaling, demonstrating potent innate immune activation [4]. Multiple antigen-only preparations, including formalin-fixed tachyzoites and tachyzoite lysate antigens, stimulated macrophage phagocytosis, increased cytotoxic leukocyte activity, and suppressed lymphoma, sarcoma, and Lewis lung carcinoma growth [17,18,19,20,21,22].

These findings were also confirmed in subsequent studies in the last two decades. TLA slowed tumor growth in fibrosarcoma, potentially through immune responses [23]. Dendritic cell-based *T. gondii* vaccines increased IL-12 and CD8^+^ activation in fibrosarcoma [24]. Attenuated ΔCPS-mutant strains robustly reversed tumor-associated immunosuppression, reactivated anergic CD8^+^ T cells, and induced durable tumor regression in ovarian, melanoma, and pancreatic tumors [25,26,28,29]. Purified *Toxoplasma* protein fractions increased pro-inflammatory Th1 immune responses and slowed melanoma growth [27]. Engineered macrophages expressing GRA15II were polarized toward antitumor M1 phenotype and inhibited hepatocellular carcinoma by modulating cytokine production (the M1 phenotype macrophages released tumor-suppressing cytokines, e.g., TNF-a and IL-12, and reduced pro-tumor factors, e.g., IL-10, IL-6, TGF-b and VEGF) [30].

DC-derived exosomes from ME49-strain-infected DCs induced STAT1-dependent M1 polarization via microRNA miR-155-5p signaling [37]. Recombinant rGRA6Nt elicited strong tumor-specific CD8^+^ responses in colorectal cancer [9]. Soluble tachyzoite antigens (STAg) and recombinant profilin (rPRF) antigens increased CD4^+^/CD8^+^ tumor infiltration and reduced regulatory T cells (Tregs) in pancreatic cancer (via toll-like receptors TLR11/12) [33].

Several attenuated *T. gondii* mutants, including Δldh, Δompdc, Δgra5, Δgra17, and non-replicating uracil auxotroph strains (NRUA), enhanced Th1 immunity, increased CD4^+^/CD8^+^ infiltration in the TME, or decreased myeloid-derived suppressor cells (MDSCs), and improved tumor control in melanoma, breast cancer, colorectal cancer, and pancreatic cancer [10,32,34,35,36,37,38]. Radiation-attenuated ME49 strain further increased IL-12 and CD8^+^ infiltration [31].

*T. gondii* infection elicited pro-inflammatory Th1 immune response, elevated levels of IFN-γ, and T cell infiltration within the glioma brain tumor, overcoming the immune suppression of the TME [3].

These findings indicate that *T. gondii* infection and parasite antigens thereof consistently reprogrammed the TME toward pro-inflammatory Th1 immunity and reversed immunosuppressive signaling.

### 3.3. Antitumor Effect via Apoptosis Induction

Three studies (6%) demonstrated direct induction of tumor-cell apoptosis independent of immune mechanisms. TLA induced apoptosis and effectively blocked neoplastic growth of human glioma cells [39]. Acute wild RH-strain infection induced rapid apoptosis in HER2/Neu^+^ breast carcinoma cells [40]. Attenuated ME49 strain activated intrinsic apoptotic pathways via marked increased expression of pro-apoptotic Bax, Bak and cytochrome-c regulators, accompanied by a significant increase in caspase-3 activity (an apoptosis performer), leading to reduction in tumor burden in Ehrlich ascites carcinoma (pro-apoptotic Bax/Bak, once activated, form pores in the outer membrane of the mitochondria (MOMP), induce MOMP permealization, release cytochrome c and activate pro-apoptotic caspase 9 and caspase 3) [41].

These findings indicate that *T. gondii* infection or parasite antigens thereof can directly initiate caspase-dependent mitochondrial apoptosis in tumor cells.

### 3.4. Antitumor Effect via Anti-Angiogenic Activity

Five studies (9%) documented reduced tumor vascularization after *T. gondii* exposure. Acute RH-strain infection decreased tumor vascularization in melanoma and lung carcinoma via elimination of VEGF expression and micro-vessel formation [42]. Acute infection induced antitumor activity in Lewis lung carcinoma through inhibition of angiogenesis (in addition to induction of Th1 immune responses via increase in CD8^+^ T cells, IFN-γ mRNA expression, and cytotoxic T-lymphocyte (CTL) responses) [43]. TLA showed an antitumor effect in sarcoma-180 via a reduction in CD31^+^ expression (a marker associated with angiogenesis) and an increase in IL-12 levels (a cytokine that suppresses tumor vascularization), in addition to activation of innate immunity [44,45]. Autoclaved *Toxoplasma* vaccine (ATV) showed antitumor activity in Ehrlich carcinoma via anti-angiogenesis by reducing VEGF (in addition to an increase in CD8^+^/T regulatory cell (Treg) ratios) [46].

These findings indicate that anti-angiogenic effects represent a reproducible, cytokine-linked mechanism of tumor suppression.

### 3.5. Antitumor Effect via Molecular Mimicry

Two studies (4%) identified antigenic cross-reactivity and the presence of shared epitopes between *T. gondii* antigens and cancer cells. Serum from *T. gondii*-infected mice selectively bound tumor cells but not normal lymphocytes, suggesting specific antigen recognition mediated by infection-induced antibodies [47]. ATV shared protein bands with Ehrlich carcinoma, indicating molecular mimicry and cross-reactive immune recognition (in addition to eliciting enhanced CD8^+^/Treg responses with reduced VEGF expression) [48].

These findings support molecular mimicry as a contributing mechanism for parasite-induced tumor control.

### 3.6. Antitumor Effect via Cell-Intrinsic Pathways

Eight studies (15%) demonstrated direct modulation of tumor-intrinsic signaling pathways by *T. gondii* or its effector proteins. Acute RH-strain infection suppressed hepatocarcinoma cancer growth by altering cell cycle regulators and reducing cell proliferation (G0/G1 cycle arrest via downregulation of cell cycle related genes *cyclin B1* and *cdc2*), in addition to promoting pro-apoptotic pathways (via an increase in caspase-3 protein and a decrease in anti-apoptotic Bcl-2 protein) [49]. Acute ME49-strain infection induced apoptosis in human T cell leukemia through the NF-kB pathway and related regulatory proteins via upregulation of A20 (a protease with indirect proapoptotic effects that inhibit NF-kB activation through an A20-mediated downregulation of antiapoptotic protein ABIN) [50]. Recombinant ROP16 induced apoptosis via the mitochondria-dependent p53 pathway (via direct serine 15/37 phosphorylation of p53), increasing pro-apoptotic Bax expression and caspase 9 and inducing cell cycle arrest in G1 phase (by increasing p21 and decreasing the CDK expression), thus promoting apoptosis in neuroblastoma [5]. Recombinant ROP16 inhibited lung adenocarcinoma cell proliferation by activating STAT3; induced cell cycle arrest at the G1 phase by increasing p21 (a known inhibitor of the cell cycle); and reduced invasion and migration of cancer cells by regulating p53, Bax, Bcl-2, cleaved-caspase 3 and caspase 9 [52]. A recombinant GRA8-derived peptide induced mitochondria activation in colon cancer (mitochondrial “metabolic resuscitation”, restoration of mitochondrial metabolic health to a state that triggers cancer cell death), leading to apoptosis [51]. Recombinant GRA16 stabilized PTEN and activated p53 tumor suppressive pathways in multiple tumors [6]. Moreover, rGRA16 inhibited AKT/NF-κB pathways and induced G2/M cell cycle arrest and apoptosis in non-small cell lung cancer [7]. Recombinant GRA16 also inhibited AKT/STAT3/NF-κB pathways and telomerase activity in colorectal cancer, causing telomere shortening and cell cycle arrest [8].

These findings demonstrate that acute infection and parasite effector proteins also engage cell-intrinsic tumor-suppressor networks independently of immune activation.

### 3.7. Pro-Tumor Effects

Two studies (4%) identified infection-induced pathways that promote tumor survival. Exosomal microRNA (miR-21) from *T.* gondii-infected microglia cells downregulated tumor-suppressor genes and enhanced glioma growth [12]. ROP18 expression after *T. gondii* infection inhibited mitochondrial apoptosis in glioblastoma cells by blocking P2X1 signaling [13].

These studies highlight that under specific contexts, particularly chronic infection or expression of certain virulence factors of *T. gondii*, *T. gondii* can promote tumor progression.

### 3.8. Dual/Stage-Dependent Divergent Effects

One study (2%) directly compared acute vs. chronic infection and demonstrated opposing effects. Acute RH-strain infection inhibited tumor growth by increasing Th1 and cytotoxic CD8^+^ cells in the TME (with higher levels of IFN-γ and other Th1-type immune responses); whereas chronic ME49-strain infection enhanced TME immunosuppression by decreasing cytotoxic CD8^+^ cells and Th1 cells infiltrations in the TME [11].

This divergence underscores that infection stage and cytokine milieu fundamentally determine the tumor outcome.

### 3.9. Signaling Pathway Transcriptomic Analysis Post-Infection Pertinent to Cancer

Five experimental studies (9%) identified signaling pathways pertinent to cancer after *T. gondii* infections in experimental non-tumor or tumor models.

*T. gondii* ME49-strain infection in neuronal cells upregulated PTEN/p53 and suppressed oncogenic pathways [53]. Infection with low-virulent strain (Prugniaud strain) downregulated oncogenic microRNAs and increased Lats1/Lats2/TNFrs11b tumor-suppressive signals [56]. *T. gondii* infection induced an antitumor effect via upregulation or downregulation of several genes respectively, including genes involved in the p53 signaling pathway, colorectal cancer pathway (e.g., DCC, Smad2, Smad 4, hMLH1, hMSH2, HMSH3), non-small cell lung cancer (NSCLC) signaling pathway (e.g., RASSF1, EGFR) and breast cancer pathway (e.g., BRCA1, CCND1) [54]. Acute *T. gondii* infection modulated miRNA expression profiles in brain tissues and modulated host cell signaling pathways regulating tumorigenesis pathways [55]. *T. gondii* infection showed an antitumor effect via transcriptional regulation of several signaling pathways related to growth and metabolism, such as the ribosome and IL-17 signaling pathways [57].

## 4. Discussion

This systematic review integrates five decades of experimental work evaluating how *Toxoplasma gondii* modulates tumor biology. Acute infection and exposure to certain parasite antigens thereof consistently elicited antitumor responses driven by IL-12- and IFN-γ-dependent pro-inflammatory Th1 immune responses, activation of macrophages and cytotoxic lymphocytes, reversal of TME immunosuppression, induction of apoptosis, and inhibition of tumor angiogenesis. Antitumor activity was also mediated by molecular mimicry and by direct modulation of cell-intrinsic signaling pathways pertinent to cancer, including stabilization of tumor-suppressor proteins, activation of p53-dependent apoptosis, inhibition of AKT/STAT3/NF-κB signaling, and reduction in telomerase activity. Recombinant parasite proteins, such as the rhoptry protein ROP16 and the dense-granule proteins GRA5, GRA6Nt, GRA-8-derived peptide, GRA15II, GRA16, and GRA17, similarly restored antitumor pathways through immune-mediated and cell-intrinsic mechanisms. Conversely, chronic infection and exposure to ROP18 effectors and exosomal microRNA miR21 exerted pro-tumor effects through enhancement of TME immunosuppression and impaired apoptosis.

Together, these findings demonstrate that *T. gondii* influences tumor biology through multiple convergent mechanisms, with acute infection and exposure to certain parasite antigens primarily supporting tumor suppression, while chronic infection and exposure to ROP18 effectors and exosomal miR21 facilitate tumor progression.

### 4.1. Antitumor Effect via Innate and Adaptive Immune Activation/Reversal of Tumor Microenvironment Immunosuppression

Acute *T. gondii* exposure—whether through live attenuated strains, inactivated preparations, or recombinant antigens—consistently elicited a strong pro-inflammatory Th1-polarized immune response characterized by IL-12 and IFN-γ production. This response activated macrophages, dendritic cells, NK cells, and cytotoxic T lymphocytes, effectively reversing tumor-associated TME immunosuppression. Parasite antigens enhanced antigen presentation, restored exhausted CD8^+^ T cell activity, and promoted M1 macrophage polarization, while also reducing the abundance and function of Tregs and myeloid-derived suppressor cells (MDSC). Collectively, these immune-mediated changes reconfigured the tumor microenvironment toward efficient immune recognition and elimination of malignant cells.

Enhancements of immune pathways are now clinically available tools for the treatment of solid tumors (e.g., via checkpoint inhibitors).

### 4.2. Antitumor Effect via Apoptosis

*T. gondii* antigens also induced apoptosis directly within tumor cells through cell-intrinsic mechanisms. Parasite-derived molecules activated intrinsic apoptotic pathways, including caspase cleavage, mitochondrial depolarization, and p53–Bax signaling cascades, independent of immune activation. These findings indicate that *T. gondii* can exert direct cytotoxic pressure on malignant cells via tumor-intrinsic apoptotic programs, representing a complementary antitumor mechanism that operates alongside—but independently from—immune-mediated effects.

### 4.3. Antitumor Effect via Anti-Angiogenic Activity

A consistent mechanistic theme across experimental models was the inhibition of tumor angiogenesis. Acute infection and parasite antigen exposure reduced tumor vascularization through downregulation of VEGF, suppression of endothelial markers of angiogenesis such as CD31 and an increase in IL-12 cytokine suppression of tumor vascularization. These anti-angiogenic effects reflected direct interference with pathways required for neovascular development. Limiting tumor blood supply and reversing VEGF-induced immunosuppression in the tumor microenvironment likely contributed to impaired tumor progression and enhanced susceptibility to immune-mediated control.

Inhibition of excess of VEGF in the tumor microenvironment via anti-VEGF monoclonal antibodies is now a strategy that is clinically available in the treatment of solid tumors. An excess of VEGF in the tumor microenvironment, in addition to the angiogenesis effect, also leads to net immunosuppression favoring tumor growth.

### 4.4. Antitumor Effect via Molecular Mimicry

A smaller but notable body of evidence indicates that *T. gondii* shares antigenic epitopes with tumor cells, enabling cross-reactive immune responses. Inactivated parasite preparations elicited antibodies and T cell responses recognizing both parasite antigens and tumor-associated targets. This molecular mimicry may broaden the antitumor repertoire by mobilizing immunity against conserved or structurally similar epitopes, contributing to the observed tumor regression in models treated with non-viable tachyzoite preparations.

### 4.5. Antitumor Effect via Cell-Intrinsic Pathways

Acute *T. gondii* infections induced apoptosis by increasing apoptosis-associated caspase-3, decreasing anti-apoptotic proteins (bcl-2), upregulating protease A20 (that inhibits NF-kB activation), and downregulating anti-apoptotic proteins (ABIN).

Moreover, *T. gondii* effector proteins, beyond immune activation, directly modulated tumor-intrinsic signaling pathways implicated in antitumor activity and apoptosis. Recombinant proteins such as GRA8-derived peptide, GRA16, and ROP16 engaged tumor-suppressive networks through diverse mechanisms, including PTEN stabilization, activation of p53 tumor suppressive pathways, p21 activation of cell-cycle arrest, inhibition of AKT/STAT3/NF-κB signaling, reduction in telomerase activity with telomere shortening, and mitochondria “metabolic resuscitation”. These cell-intrinsic effects reflect the capacity of parasite molecules to reprogram oncogenic circuits, steering tumor cells toward apoptosis, growth arrest, or loss of proliferative capacity.

### 4.6. Pro-Tumor Effects

While antitumor effects predominated in acute or antigen-based models, chronic infection and exposure to specific virulent effectors, such as ROP18, demonstrated pro-tumor activity. Persistent infection promoted inflammatory conditions favorable to tumor survival. The virulence-associated rhoptry protein ROP18 and infection-induced exosomal miR-21 impaired apoptosis and supported tumor cell proliferation.

In many tumors, microRNA-21 (miR-21) acts as an oncogenic microRNA that promotes tumor growth, survival, and treatment resistance by blocking tumor suppressors (like PTEN) and activating tumor pro-survival pathways (like PI3K/AKT) [61]. In infections, especially by oncogenic viruses (HBV, HCV, HPV), elevated miR-21 helps pathogens evade immune responses and promotes viral replication, while also influencing inflammation and immune cell function [61]. The association between dormant infections and cancer has been previously demonstrated in bladder cancer (*Schistosoma haematobium*), biliary tract cancer (*Clonorchis sinensis*), and stomach cancer (*Helicobacter pylori*).

Although both rhoptry proteins ROP16 and ROP18 are virulent factors, they do exhibit divergent actions in tumor biology. ROP18 promotes tumor cell survival by suppressing intrinsic mitochondrial apoptosis and disabling host immune defenses (like IRGs—Immunity-Related GTPases) [62], whereas ROP16 shows antitumor effects through activation of p53/p21-linked tumor-suppressive signaling. These findings underscore the critical importance of proper antigen selection for future *T. gondii* cancer immunotherapy applications.

### 4.7. Dual/Stage-Dependent Divergent Effects

Some models revealed opposing effects depending on infection stage. Acute infection triggered robust Th1 immunity and tumor suppression, whereas chronic infection enhanced TME immunosuppression by decreasing cytotoxic CD8^+^ cell and Th1 cell infiltration and facilitated tumor progression. This divergence illustrates a fundamental principle: *T. gondii*-mediated effects on tumor biology depend on the balance between early infection associated pro-inflammatory activation and late infection associated immunoregulatory or persistence-associated responses.

### 4.8. Signaling Pathways Pertinent to Cancer

Certain experimental studies described wide-ranging transcriptomic and microRNA alterations induced by parasite exposure, influencing pathways involved in apoptosis, autophagy, oxidative stress, immune regulation, and oncogenic signaling. These mixed or global effects suggest that *T. gondii* can exert multilayered pressure on the tumor environment, with impacts not limited to a single mechanistic pathway. Such broad reprogramming may help explain the diversity of antitumor responses observed across models and tumor types.

### 4.9. Study Limitations

In this systematic review, studies varied widely in parasite strains, infection routes, tumor models, antigen preparations, and outcome measurements thereof, limiting direct comparability and precluding quantitative data synthesis and meta-analysis of antitumor and pro-tumor effects of *T. gondii*. In a few instances, detailed mechanistic interpretation was limited by incomplete molecular dissection of the signaling pathways underlying the observed phenotypes. The literature search was conducted in a single bibliographic database (PubMed), and although additional manual reference screening was performed, some relevant studies indexed exclusively in other databases or gray literature sources may not have been captured.

### 4.10. Translational and Therapeutic Implications

This systematic review identified strong experimental evidence supporting the antitumor effects of inactivated *T. gondii* strains, attenuated *T. gondii* mutants and select recombinant *T. gondii* proteins. Attenuated non-replicating parasite strains, as well as recombinant parasite proteins with reproducible antitumor activity such as the rhoptries protein ROP16 and the dense-granule proteins GRA5, GRA6Nt, GRA8-derived peptide, GRA15II, GRA16, and GRA17, demonstrated immune-mediated and cell-intrinsic tumor-suppressive mechanisms across multiple experimental models. Non-replicating and attenuated *T. gondii* strains, including uracil auxotroph mutants, Δldh1/Δldh2, Δgra17, Δgra5, and radiation-attenuated ME49 strains, consistently induced strong IL-12/IFN-γ responses while avoiding uncontrolled parasite replication.

Recombinant parasite proteins with reproducible antitumor activity recapitulated key immune-mediated and cell-intrinsic tumor-suppressive pathways observed with live wild-type or attenuated infections. These proteins modulated Th1 immunity, macrophage polarization, and tumor-intrinsic signaling pathways such as PTEN–p53 and AKT/STAT3/NF-κB, without the safety risks associated with replicating parasites.

Such antigen-focused strategies resemble the conceptual framework of bacillus Calmette–Guérin (BCG) microbial cancer immunotherapies and highlight the potential of *T. gondii*-derived platforms for cancer immunotherapy as well. Select *T. gondii* antigens with high immunogenic and antitumor potential should also be studied in mRNA vaccine platforms as candidates for clinical development beginning with animal models. Monitoring cytokine profiles to demonstrate enhancing of pro-inflammatory cytokines and decreasing of anti-inflammatory cytokines could potentially be used as a surrogate marker for vaccine efficacy. Future translational efforts should prioritize rigorous clinical evaluation of the safety and efficacy of non-replicating *T. gondii* strains and recombinant *T. gondii* proteins with validated antitumor potential. They may be particularly promising in immunologically “cold” tumors such as glioblastoma [63,64]. However, the pro-tumor potential of the rhoptry protein ROP18 underscores the need for precise antigen selection and rigorous safety evaluation. In addition, epidemiological studies suggesting a higher *Toxoplasma* IgG seroprevalence in patients with cancer should be followed with registries addressing whether the cancer prognosis (e.g., progression-free survival and overall survival under treatment) is adversely affected by *Toxoplasma* infection status.

## 5. Conclusions

This systematic review integrated five decades of experimental work and identified evidence supporting the antitumor effect of *T. gondii* after acute infections (including infections with non-replicating attenuated or mutant *T. gondii* strains) and after exposure to certain parasite antigens, recombinant proteins (e.g., rhoptry protein ROP16 and dense-granule proteins GRA5, GRA6Nt, GRA-8-derived peptide, GRA15II, GRA16, and GRA17), and DC-derived exosomal microRNAs (miR-155-5p). The dominant implicated mechanistic pathways for the antitumor effects were the activation of innate and adaptive immunity, reversal of tumor microenvironment immunosuppression, induction of apoptosis, inhibition of angiogenesis, molecular mimicry, and modulation of cell-intrinsic pathways. Conversely, pro-tumor effects were seen with chronic infection and exposure to ROP18 effector protein and miR-21. Future translational studies for *T. gondii*-based cancer immunotherapeutic approaches should prioritize attenuated non-replicating *T. gondii* strains and certain recombinant parasite antigens with demonstrated antitumor activity, including also development of mRNA vaccine platforms, with emphasis on rigorous evaluation of their safety and efficacy.

## Figures and Tables

**Table 1:** Characteristics of the experimental studies included in the systematic review (*n* = 54).

Study Characteristic	N of Articles (*n* = 54)
**Publication years**	1971–2024
**Experimental studies**	**54**
In vivo only models	41
In vitro only models	10
Both in vitro and in vivo models	3
**In vitro cancer cell line models ^1^**	**13**
Glioma/glioblastoma	3
Colorectal cancer	3
Breast cancer	1
Hepatocarcinoma	1
Fibrosarcoma	1
Melanoma	1
Lung adenocarcinoma	1
Neuroblastoma	1
Human T cell leukemia	1
**In vivo animal cancer models ^2^**	**44**
Sarcoma/fibrosarcoma	9
Breast cancer	6
Colorectal cancer	6
Lung cancer (e.g., Lewis lung carcinoma)	6
Melanoma	6
Glioma/glioblastoma	4
Pancreatic cancer	4
Hepatocarcinoma	3
Feline lymphoma/lymphoma	3
Ehrlich ascites carcinoma	2
Ehrlich solid carcinoma	2
Ovarian cancer	2
Non-small cell lung cancer	1
Leukemia (e.g., AKR, Friend, human T cell leukemia)	1
Neuroblastoma	1
Ependymoblastoma	1
***T.gondii *** **(strain/antigens/recombinant proteins)**	
Wild-type strain *T. gondii* infections (virulent type I RH strain, less virulent type II ME49, Prugniaud, Beverley strain, type III VEG strain, and YZ strain)	16
Attenuated/inactivated strain *T. gondii* infections (mutant strains: Δcps, Δldh, Δompdc, Δgra5, Δgra17; NRUA; radiation-attenuated or autoclaved strains)	13
Parasite antigen exposure (TLA, STAgs, formalin-fixed tachyzoites)	12
Parasite antigens + recombinant protein	1
Exosomes (from TLA-pulsed or *T. gondii*-infected DC-derived exosomes)	3
Recombinant *T. gondii* proteins (rROP16, rROP18, rGRA5, rGRA6Nt, rGRA8, rGRA15II, rGRA16)	8
Not applicable (Serum from infected mice for molecular mimicry study)	1
**Stage of ** ***T. gondii*** **infection**	
Acute *T. gondii* infection (animal tumor models: *n* = 19) (Type I, II or III strains: *n* = 6; attenuated, inactivated or mutant strains: *n* = 13)	19
Acute vs. chronic *T. gondii* infection (animal tumor model: *n* = 1)	1
Exposure to non-infectious *T. gondii* material (TLA, exosomes, recombinant proteins) (animal tumor models: *n* = 18, both in vitro/in vivo tumor models: *n* = 3)	21
Stage of infection not pertinent—only in vitro tumor model (in vitro *T. gondii* infection: *n* = 2; in vitro exposure to non-infectious material: *n* = 5, in vitro *T. gondii* infection and exposure to non-infectious material: *n* = 1)	8
Signaling pathway/transcriptomic analysis pertinent to carcinogenesis post-infection (in vivo acute infection in non-tumor model: *n* = 3; in vitro infection in non-tumor model: *n* = 1; in vitro infection in tumor model: *n* = 1)	5
**Cancer effect**	
Antitumor effect	46
Pro-tumor effect	2
Dual stage-dependent divergent effect (acute infection/antitumor vs. chronic infection/pro-tumor)	1
Signaling pathway/transcriptomic analysis pertinent to cancer post-infection (non-tumor models: *n* = 4; tumor model: *n* = 1)	5
**Mechanistic categories**	
Antitumor effect via innate or adaptive immune activation/reversal of TME immunosuppression	28
Antitumor effect via apoptosis	3
Antitumor effect via anti-angiogenesis	5
Antitumor effect via molecular mimicry	2
Antitumor effect via cell-intrinsic pathways	8
Pro-tumor effect	2
Dual stage-dependent divergent effect (acute infection/antitumor vs. chronic infection/pro-tumor)	1
Signaling pathway/transcriptomic analysis pertinent to cancer post-infection (non-tumor models *n* = 4, tumor model *n* = 1)	5

^1^ The total listed in vitro cancer models is *n* = 13 (10 in vitro-only studies + three both in vitro/in vivo); ^2^ the total listed in vivo cancer models exceeds 44 (41 in vivo-only + three both in vivo/in vitro) as some studies have used more than one models. Abbreviations: DC, dendritic cell; NRUA, non-replicating uracil auxotroph strain; rROP, recombinant rhoptry protein; rGRA, recombinant dense granule protein; STAg, soluble tachyzoite antigen; TLA, tachyzoite lysate antigen; TME, tumor microenvironment.

**Table 2:** Mechanistic categories with respect to *T. gondii* effects in cancer biology (*n* = 54).

Study	Infection Type/Experimental Model	Mechanistic Effect
**Antitumor Effect—Via Innate and Adaptive Immune Activation/Reversal of Tumor Microenvironment (TME) Immunosuppression ^1,2,3^**		
Hibbs & Remington 1971 [1]	Sulfadiazine-treated acute *T. gondii* infection; mammary carcinoma, AKR leukemia, Friend leukemia, leukemia, and sarcoma models	Sulfadiazine-treated acute *T. gondii* infection activated macrophage-driven nonspecific cytotoxicity (through a nonphagocytic mechanism), delaying and reducing tumor growth.
Conley 1977 [2]	Sulfadiazine-treated acute *T. gondii* infection (type I RH strain); intracerebral ependymoblastoma mouse model	Sulfadiazine-treated acute *T. gondii* infection recruited activated microglia/macrophages that restricted intracerebral tumor expansion.
Kamiyama 1984 [4]	Acute type II Beverley strain infection; NK-deficient mouse lymphoma model	Acute *T. gondii* infection restored NK cytotoxicity via IFN-dependent signaling.
Suzuki 1985 [17]	Parasite antigens (formalin-fixed tachyzoites); Lewis lung carcinoma mouse model	Formalin-fixed tachyzoites altered macrophage responses and tumor behavior through delayed-type hypersensitivity stimulation, inhibiting tumor growth.
Suzuki 1986 [18]	Parasite antigens (formalin-fixed tachyzoites); EL-4 lymphoma model	Antigen exposure activated macrophage and NK responses, suppressing lymphoma growth.
Saito 1989 [19]	Parasite antigens (TLA); sarcoma mouse models (S-180, Meth A)	TLA enhanced macrophage phagocytosis and inhibited tumor growth.
Yang 1990 [20]	Parasite antigens (TLA); feline lymphoma co-culture model	TLA increased cytotoxic leukocyte activity and interferon-like responses.
Miyahara 1992 (mouse model) [21]	Parasite antigens (TLA); methylcholanthrene-induced mouse sarcoma model	Repeated TLA exposure enhanced macrophage/lymphocyte cytotoxicity and reduced tumor volume.
Miyahara 1992 (rat model) [22]	Parasite antigens (TLA); methylcholanthrene-induced rat sarcoma model	TLA delayed tumor growth and boosted systemic cytotoxic immune responses.
Darani 2009 [23]	Parasite antigens (TLA); WEHI-164 fibrosarcoma mouse model	TLA slowed tumor growth potentially mediated through immune responses.
Motamedi 2009 [24]	Parasite antigen (TLA-pulsed dendritic cell vaccine); fibrosarcoma mouse model	TLA injection in dendritic cells enhanced IL-12 production and CD8^+^ activation.
Baird 2013 [25]	Attenuated ΔCPS (non-replicating uracil auxotroph strain—NRUA); ID8-VEGF ovarian cancer mouse model	ΔCPS strain reversed tumor immunosuppression via IL-12/IFN-γ and restored potent antitumor CD8^+^ T cell responses.
Fox 2013 [26]	Attenuated ΔCPS strain; melanoma and ovarian tumor mouse models	ΔCPS strain reactivated anergic T cells and induced durable tumor regression.
Boghozian 2015 [27]	Parasite antigens (STAgs) protein fractions; B16-F10 melanoma mouse model	*Toxoplasma* protein fractions (A1 and C14) activated dendritic cells, promoted Th1 immune responses, and slowed melanoma growth.
Sanders 2015 [28]	Attenuated ΔCPS strain; pancreatic adenocarcinoma mouse model	ΔCPS strain decreased tumor-associated macrophages, increased dendritic cell infiltration, boosted IL-12 production, and enhanced CD4^+^ and CD8^+^ T cell infiltration within the tumor microenvironment and activated tumor-specific CD8^+^ T cell responses.
Sanders 2016 [29]	Attenuated ΔCPS strain; metastatic pancreatic cancer mouse model	ΔCPS strain induced tumor regression by increasing CD4^+^ and CD8^+^ T cell responses (overcoming the TME immunosuppression) and long-term antitumor immunity (by promoting tumor-specific IgG).
Li Y 2017 [30]	Recombinant GRA15-II protein; hepatocellular carcinoma mouse model	GRA15-II polarized macrophages toward an antitumor M1 polarized macrophages phenotype.
Hafez 2020 [31]	Radiation-attenuated type II ME49 strain; Ehrlich ascites carcinoma mouse model	Attenuated strain increased IL-12/CD8^+^ T cell responses and reduced angiogenesis markers with reversal of cancer-associated immunosuppression and reduction in tumor progression.
Li Y 2021 [32]	Attenuated Δldh mutant strain; B16F1 melanoma mouse model	Δldh mutant induced strong Th1/CD8^+^ immunity and suppressed melanoma growth.
Payne 2021 [33]	Parasite antigens (STAgs and recombinant profilin [rPRF]); KPC pancreatic cancer mouse model	STAgs increased CD4^+^/CD8^+^ infiltration and reduced regulatory T cells (Tregs) via toll-like receptor (TLR)11/12–IFN-γ signaling.
Xu 2021 [34]	Attenuated Δompdc mutant strain; 4T1 breast cancer mouse model	Δompdc mutant increased IL-12/iNOS/TNF-α and reduced metastatic spread.
Zhu YC 2021 [10]	Attenuated Δgra17 mutant strain; melanoma, colorectal carcinoma, and lung carcinoma mouse models	Δgra17 mutant strain increased innate and adaptive immune infiltration, converting immunologically “cold” tumors to “hot” tumors responsive to checkpoint blockade.
Bahwal 2022 [35]	Non-replicating uracil auxotroph strain (NRUA); Pan02 pancreatic cancer mouse model	Activated dendritic cells, enhanced CD8^+^ infiltration, and reduced myeloid-derived suppressor cells (MDSCs).
Lu 2022 [36]	Dendritic cell (DC)-derived exosomes from ME49 infection; CT26 colorectal cancer mouse model	DC-derived exosomes reduced myeloid-derived suppressor cells (MDSCs) via STAT3 inhibition.
Zhu S 2022 [37]	DC-derived exosomes from type II ME49 infection; colorectal carcinoma model	DC-derived microRNA miR-155-5p exosomal signaling induced STAT1-dependent M1 macrophage polarization.
Chen 2023 [38]	Attenuated ME49 Δgra5 mutant strain; 4T1 breast carcinoma mouse model	Δgra5 mutant strain increased CD4^+^/CD8^+^ infiltration and systemic IFN-γ/IL-12 responses.
Mani 2024 [9]	Recombinant rGRA6Nt vaccination; MC38 colorectal carcinoma mouse model	rGRA6Nt induced tumor-specific IFN-γ^+^ CD8^+^ T cells.
Nguyen 2024 [3]	Acute type II ME49 infection; murine glioma model	Acute infection elicited pro-inflammatory Th1 immune responses, elevated levels of IFN-γ, and increased T cell infiltration within the glioma tumor microenvironment, overcoming the immune suppression of the tumor microenvironment.
**Antitumor Effect—Via Apoptosis Induction ^1^**		
Choo 2005 [39]	Parasite antigen (TLA); human glioma model (in vitro and in vivo)	TLA increased tumor cell apoptosis.
Şahar 2020 [40]	Type I RH infection; HER2/Neu^+^ breast carcinoma cell model	Tachyzoite infection induced rapid apoptosis.
Hafez 2020 [41]	Radiation-attenuated type II ME49 strain; Ehrlich ascites carcinoma mouse model	Attenuated strain activated intrinsic apoptotic pathways, increased IL-12/IFN-γ/CD8^+^, reduced angiogenesis markers, nitric oxide and tumor-promoting inflammatory markers (STAT-3 and TNF-a) and reduced tumor burden.
**Antitumor Effect—Via Anti-Angiogenesis ^1^**		
Hunter 2001 [42]	Acute type I RH infection; B16 melanoma and Lewis lung carcinoma mouse models	Acute infection eliminated VEGF expression and tumor vascularization.
Kim 2007 [43]	Acute type II ME49 infection; Lewis lung carcinoma mouse model	Acute infection induced Th1 immune responses and anti-angiogenic activity.
Pyo 2010 [44]	Parasite antigens (TLA); Sarcoma-180 mouse model	TLA reduced CD31 expression (an angiogenesis associated marker) and increased IL-12 levels (a tumor vascularization suppressing cytokine).
Pyo 2014 [45]	Parasite antigens (TLA); Sarcoma-180 nude mouse model	TLA induced IL-12 production and activated macrophage-mediated innate immune responses independent of adaptive immunity.
Ismail 2023 [46]	Autoclaved *Toxoplasma* vaccine (ATV) ± cyclophosphamide; Ehrlich solid carcinoma mouse model	ATV reduced VEGF and increased CD8^+^/Treg ratios inside the tumor.
**Antitumor Effect—Via Molecular Mimicry**		
Mohamadi 2019 [47]	Serum from *T. gondii*-infected mice; murine tumor cell lines (fibrosarcoma, melanoma)	Antibodies from infected mice selectively bound tumor cells, indicating antigenic mimicry and tumor-selective immune recognition.
Eissa 2023 [48]	Autoclaved *Toxoplasma* vaccine (ATV); Ehrlich solid carcinoma mouse model	ATV shared antigenic protein bands between parasite and tumor tissue indicating molecular mimicry and cross-reactive immune recognition.
**Antitumor Effect—Via Cell-Intrinsic Pathways ^1^**		
Chang 2015 [5]	Recombinant ROP16 protein; neuroblastoma cell model	rROP16 promoted apoptosis by activating p53 signaling (which directly activates the proapoptotic Bax protein) and induced G1-phase cell cycle arrest by increasing p21 expression and decreasing CDK expression.
Wang 2016 [49]	Acute type I RH infection; hepatocarcinoma cell model	Acute infections induced apoptosis through G0/G1 cell-cycle arrest, caspase-3 activation and decrease in the anti-apoptotic Bcl-2 protein.
Chen 2018 [50]	Acute type II ME49 infection; human leukemia T cell model	Acute infection induced apoptosis via upregulation of A20 protease (that inhibits NF-κB signaling) and downregulation of antiapoptotic protein ABIN.
Kim 2019 [6]	Recombinant GRA16 protein; HepG2 tumor model	rGRA16 stabilized PTEN and activated p53 tumor-suppressive pathways.
Seo 2020 [7]	Recombinant GRA16 protein; non-small cell lung carcinoma (NSCLC) mouse model	rGRA16 inhibited AKT/NF-κB signaling and induced G2/M arrest and apoptosis, reducing chemoresistance (via inhibition of NF-kB activation).
Kim 2020 [51]	Recombinant GRA8-derived peptide; HCT116 colon cancer in vitro and in vivo model	rGRA8-derived peptide induced mitochondrial-mediated apoptosis via mitochondria activation (mitochondria “metabolic resuscitation”).
Seo 2022 [8]	Recombinant GRA16 protein; colorectal cancer cell model	rGRA16 stabilized the tumor suppressor PTEN, inhibited AKT/STAT3/NF-κB signaling, and caused telomere shortening, DNA damage, and cell cycle arrest, ultimately reducing tumor proliferation.
Li G 2024 [52]	Recombinant ROP16 protein; lung adenocarcinoma models	rROP16 activated STAT3 (inhibiting cancer cell proliferation); increased p21 (inducing cell cycle arrest); regulated p53, Bax/Bcl-2, cleaved-caspase 3 and caspase 9 (reducing the invasion and migration of cancer cells) and induced apoptosis.
**Pro-Tumor Effect ^4^**		
Zhou 2019 [13]	*T. gondii* infection (Type I strain RH, RH-∆rop18, RH-ROP18-GFP-Flag, Type II strain ME49, Type III strain VEG), ROP18 expression; U251 glioblastoma model	ROP18 expression after *T. gondii* infection inhibited ATP-induced apoptosis by interfering with P2X1 function and the mitochondrial apoptotic pathway.
Jung 2022 [12]	Exosomal microRNA miR-21 from *T. gondii* (Type I-RH and Type II-ME49)-infected BV2 microglia cells; U87 glioma model	Infection-derived exosomal miR-21 suppressed tumor-suppressor genes (FoxO1, PTEN, and PDCD4) and promoted tumor growth.
**Dual/Stage-Dependent Divergent Effects**		
Song 2024 [11]	Acute type I RH infection vs. chronic type II ME49 infection; Lewis lung cancer mouse model	Acute RH-strain infection inhibited tumor growth by increasing Th1 and cytotoxic CD8^+^ cells in the TME (inducing higher levels of IFN-γ and other Th1-type immune responses); whereas chronic ME49-strain infection enhanced TME immunosuppression by decreasing cytotoxic CD8^+^ cell and Th1 cell infiltration.
**Signaling Pathway/Transcriptomic Analyses Pertinent to Cancer ^5^**		
Ngô 2017 [53]	Signaling pathway analysis/disease-deconvolution computational analysis after *T. gondii* infection (Type I, II, III) of brain cells; non-tumor infection model/signaling pathway analysis	Computational signaling pathway analysis of *T. gondii* infected cells revealed modulation of important cancer-related pathways (e.g., Wnt/Ca + pathway (14 genes), TGF-β, and STAT pathway)
Lu 2019 [54]	Acute type II (Prugniaud strain) infection of mice; non-tumor infection model/signaling pathway analysis	Transcriptomic analysis of mRNA after mice acute *T. gondii* infection showed changes in cancer pathways towards inhibition of tumor growth; via upregulation or downregulation towards the antitumor effect of genes involved in the p53 signaling pathway, colorectal cancer pathway (DCC, Smad2, Smad 4, hMLH1, hMSH2, HMSH3), non-small cell lung cancer signaling pathway (RASSF1, EGFR) and breast cancer pathway (BRCA1, CCND1).
Hou 2022 [55]	Acute *T. gondii* infections (YZ-1 strain) of porcine brain; non-tumor infection pig model/signaling pathway analysis via miRNA profiling	Acute *T. gondii* infection modulated miRNA expression profiles in brain tissues and modulated host cell signaling pathways regulating cancer-related pathways (in addition to apoptosis, NF-κB inflammatory response, autophagy, oxidative stress).
Wang 2022 [56]	Acute infection of mice with low virulent type II *T. gondii* strain (Prugniaud strain); non-tumor infection model/signaling pathway analysis via miRNA transcriptomic analysis	Acute *T. gondii* infection of mice suppressed tumor growth through miRNA-mediated pathways, downregulated oncogenic miRNAs, and increased expression of tumor suppressor genes Lats1/Lats2/TNFrs11b.
Ye 2024 [57]	Type I (RH) and Type II (ME49) strain infection; human breast cancer in vitro infection model/signaling pathway analysis	*T. gondii* infection showed an antitumor effect via transcriptional regulation of several signaling pathways related to growth and metabolism, such as the ribosome and IL-17 signaling pathways.

^1^ Studies were categorized according to their primary experimentally demonstrated mechanism. Studies describing multiple mechanistic pathways were assigned to the dominant or most extensively validated mechanistic category in the study. Some studies may have contributed mechanistic insights in more than one context. ^2^ The broad mechanistic group categories were defined a priori; the exact mechanisms in each category were determined based on the actual identified experimental data and pertained to the following: (a) innate or adaptive immune activation/reversal of tumor microenvironment (TME) immunosuppression (e.g., via macrophage/NK activation, dendritic cell priming, Th1 response activation, macrophage polarization to antitumor M1 phenotype); (b) induction of apoptosis (e.g., via caspase activation of mitochondrial apoptosis); (c) anti-angiogenesis (e.g., VEGF suppression/reduced vascularization); (d) molecular mimicry (cross-reactivity between *T. gondii* antigens and tumor antigens); (e) antitumor cell-intrinsic pathways (e.g., via modulation of PTEN/p53/p21 signaling, inhibition of AKT, STAT3, NF-κB, telomerase shortening); (f) pro-tumor effect (e.g., exosomal microRNA 21 decreased expression of tumor suppressor genes; ROP18 expression inhibited apoptosis); (g) dual/stage-dependent divergent effects (acute infection: antitumor effect via reversal of TME immunosuppression vs. chronic infection: pro-tumor effect via enhanced TME immunosuppression); (h) signaling pathway/transcriptomic analysis post-infection pertinent to cancer. ^3^ It is acknowledged that there is overlap between the above mechanistic categories and some studies could have been categorized under more than one mechanistic category; however, for the purposes of this systematic review, each study was categorized under the dominant mechanistic category described in the study. Additional mechanistic details for each study are included in Supplementary Table S1. ^4^ Included here are experimental studies describing direct pro-tumor effects in experimental tumor models. ^5^ Included here are experimental studies using signaling pathway/transcriptomic analyses post-*T. gondii* infections describing signaling pathways pertinent to cancer effects (in experimental non-tumor or tumor models). Abbreviations: ATV, autoclaved *Toxoplasma* vaccine; DC, dendritic cell; IFN-γ, interferon-gamma; IL-, interleukin-; Lats: large tumor suppressor gene; MDSC, myeloid-derived suppressor cell; miRNA, microRNA; NF-κB, nuclear factor kappa-light-chain-enhancer of activated B cells; NK, natural killer; NRUA, non-replicating uracil auxotroph; rROP-, recombinant ROP; rGRA-, recombinant GRA; STAg, soluble tachyzoite antigens; TLA, tachyzoite lysate antigens; TME, tumor microenvironment; TNFrs, tumor necrosis factor receptor superfamily gene; Treg, regulatory T cell; VEGF, vascular endothelial growth factor.

## Data Availability

All data for this project are available in the main paper, supporting Appendix A and Appendix B and Supplementary Material.

## Supplementary Materials

The following supporting information can be downloaded at: https://www.mdpi.com/article/10.3390/pathogens15040351/s1, Table S1: Data extraction–mechanistic details per study (vignettes).

## Abbreviations

The following abbreviations are used in this manuscript:

AKT	Protein kinase B
ATV	Autoclaved *Toxoplasma gondii* vaccine
BCG	Bacillus Calmette–Guérin
Bcl-2	B-cell lymphoma 2
CD	Cluster of differentiation
CNS	Central nervous system
DC	Dendritic cell
EGFR	Epidermal growth factor receptor
GRA	Dense granule protein
HBV	Hepatitis B virus
HCV	Hepatitis C virus
HPV	Human papillomavirus
IFN-γ	Interferon gamma
IL	Interleukin
IRG	Immunity-related GTPase
Lats	Large tumor suppressor gene
MDSC	Myeloid-derived suppressor cell
miR	MicroRNA
NF-κB	Nuclear factor kappa B
NK	Natural killer
NRUA	Non-replicating uracil auxotroph
PRISMA	Preferred Reporting Items for Systematic Reviews and Meta-Analyses
OSF	Open Science Framework
PTEN	Phosphatase and tensin homolog
ROP	Rhoptry protein
STAT	Signal transducer and activator of transcription
STAg	Soluble tachyzoite antigen
TGF-β	Transforming growth factor beta
TLA	Tachyzoite lysate antigen Th1 T helper 1
*T. gondii*	*Toxoplasma gondii*
TME	Tumor microenvironment
TNFrs	Tumor necrosis factor receptor superfamily gene
Treg	Regulatory T cell
VEGF	Vascular endothelial growth factor

## Appendix A. Search Strategy

A comprehensive and systematic literature search was conducted in PubMed to identify experimental studies evaluating the antitumor or pro-tumor effects of *Toxoplasma gondii* infection or parasite-derived antigens. Searches were performed without language restrictions. However, during the eligibility screening process, only studies published in English or Spanish were included in the review to ensure accurate assessment of study methods and results. The last search was conducted on 9 September 2024. The search strategy combined parasite-related terms and cancer-related terms using Boolean operators. Given the specific interest in the potential relationship between chronic *T. gondii* infection and tumors of the central nervous system, additional searches incorporating brain tumor-specific terms were conducted to increase sensitivity for central nervous system malignancies.

To ensure completeness, the reference lists of relevant review articles and all eligible full-text studies were manually screened to identify additional experimental studies that may not have been captured through the electronic database search. The search strategy was designed to identify in vitro and in vivo experimental studies or in silico signaling pathway analyses explicitly evaluating mechanistic effects of *T. gondii* infection or parasite-derived preparations on tumor biology, including antitumor, pro-tumor, dual stage-dependent, or pro-tumor signaling effects. Epidemiologic association studies, seroprevalence studies, clinical case reports or case series, editorials, and narrative reviews were excluded. The different search strategies used in this systematic review are listed below. Non-overlapping articles were screened at the title level first, and potentially eligible studies were subsequently screened at the abstract and full-text level (as described in Figure 1).

SEARCH STRATEGIES USED:

#1 (toxoplasma* OR t.gondii OR T. gondii) AND (tumor*[ti] OR cancer*[ti] OR carcinogen*[ti] OR oncogen*[ti] OR malignan*[ti] OR antitumor [ti]) (N = 248 articles up to 9 April 2024)
#2: (toxoplasma [ti] OR t.gondii [ti] OR t. gondii[ti]) AND (brain OR neurologic OR neural OR nervous OR cerebral) AND (tumor* OR cancer* OR malignan* OR carcino* OR oncogene* OR antitumor*) (N = 153 articles up to 9 April 2024)
#3(toxoplasma* [ti] OR t.gondii [ti] OR T. gondii [ti]) AND (tumor*[ti] OR cancer*[ti] OR carcinogen*[ti] OR oncogen*[ti] OR malignan*[ti] OR antitumor [ti] OR Meningioma* OR Pituitary adenoma* OR Schwannoma* OR acoustic neuroma* OR Choroid plexus tumor* OR Dysembryoplastic neuroepithelial tumor* OR Neurofibroma* OR Hemangioblastoma* OR Giant cell tumor* OR Glioma* OR astrocytoma* OR oligodendroglioma* OR glioblastoma* OR Ependymal tumor* OR subependymoma* OR ependymoma* OR hemangiopericytoma* OR Germ Cell Tumor* OR Pineal tumor* OR Medulloblastoma* OR neuroblastoma* OR Lymphoma* OR gliosarcoma*)) (N = 237 up to 9 April 2024)
#4((toxoplasma* [ti] OR t.gondii [ti] OR T. gondii [ti]) AND (tumor* OR cancer* OR carcinogen* OR oncogen* OR malignan* OR oncolo* OR antitumor* OR anti-tumor* OR brain tumor* OR brain cancer* OR CNS tumor* OR CNS cancer* OR glioma* OR astrocytoma* OR ganglioglioma* OR xanthoastrocytoma* OR neuroepithelial tumor* OR glioneuronal tumor* OR oligodendroglioma* OR astroblastoma* OR neuronal tumor* OR gangliocytoma* OR neurocytoma* OR liponeurocytoma* OR glial tumor* OR glioblastoma* OR ependymoma* OR subependymoma* OR choroid plexus tumor* OR choroid plexus papilloma* OR choroid plexus carcinoma* OR embryonal tumor* OR medulloblastoma* OR embryonal tumor* OR neuroblastoma* OR CNS tumor* OR pineocytoma* OR pineal parenchymal tumor* OR pineoblastoma* OR tumor of the pineal* OR Schwannoma* OR neurofibroma* OR perineurioma* OR nerve sheath tumor* OR paraganglioma* OR meningioma* OR meningothelial tumor* OR Pituitary adenoma* OR acoustic neuroma* OR neurofibroma* OR hemangioblastoma* OR giant cell tumor* OR ependymal tumor* OR hemangiopericytoma* OR germ cell tumor* OR pineal tumor* OR medulloblastoma* OR neuroblastoma* OR lymphoma* OR gliosarcoma*)) (N = 868 up to 9 April 2024)
#3 NOT (#1 OR #2)
#4 NOT (#1 OR #2 OR #3)

## Appendix B. PRISMA 2020 Abstract and PRISMA 2020 Checklist

**Table A1:** PRISMA 2020 abstract.

Section and Topic	Item #	Checklist Item	Reported (Yes/No)
**TITLE**			
Title	1	Identify the report as a systematic review.	Yes
**BACKGROUND**			
Objectives	2	Provide an explicit statement of the main objective(s) or question(s) the review addresses.	Yes
**METHODS**			
Eligibility criteria	3	Specify the inclusion and exclusion criteria for the review.	Yes
Information sources	4	Specify the information sources (e.g., databases, registers) used to identify studies and the date when each was last searched.	Yes
Risk of bias	5	Specify the methods used to assess risk of bias in the included studies.	Not applicable
Synthesis of results	6	Specify the methods used to present and synthesize results.	Yes
**RESULTS**			
Included studies	7	Give the total number of included studies and participants and summarize relevant characteristics of studies.	Yes
Synthesis of results	8	Present results for main outcomes, preferably indicating the number of included studies and participants for each. If meta-analysis was done, report the summary estimate and confidence/credible interval. If comparing groups, indicate the direction of the effect (i.e., which group is favored).	Yes
**DISCUSSION**			
Limitations of evidence	9	Provide a brief summary of the limitations of the evidence included in the review (e.g., study risk of bias, inconsistency and imprecision).	Yes
Interpretation	10	Provide a general interpretation of the results and important implications.	Yes
**OTHER**			
Funding	11	Specify the primary source of funding for the review.	Yes
Registration	12	Provide the register name and registration number.	Yes

From:Page et al. (2021) [59].

**Table A2:** PRISMA 2020 checklist.

Section and Topic	Item #	Checklist Item	Location Where Item Is Reported
**TITLE**			
Title	1	Identify the report as a systematic review.	Page 1
**ABSTRACT**			
Abstract	2	See the PRISMA 2020 for abstracts checklist.	Appendix B
**INTRODUCTION**			
Rationale	3	Describe the rationale for the review in the context of existing knowledge.	Page 1 and 2
Objectives	4	Provide an explicit statement of the objective(s) or question(s) the review addresses.	Page 2
**METHODS**			
Eligibility criteria	5	Specify the inclusion and exclusion criteria for the review and how studies were grouped for the syntheses.	Page 3
Information sources	6	Specify all databases, registers, websites, organizations, reference lists and other sources searched or consulted to identify studies. Specify the date when each source was last searched or consulted.	Page 3
Search strategy	7	Present the full search strategies for all databases, registers and websites, including any filters and limits used.	Appendix A
Selection process	8	Specify the methods used to decide whether a study met the inclusion criteria of the review, including how many reviewers screened each record and each report retrieved, whether they worked independently, and, if applicable, details of automation tools used in the process.	Page 3
Data collection process	9	Specify the methods used to collect data from reports, including how many reviewers collected data from each report, whether they worked independently, any processes for obtaining or confirming data from study investigators, and, if applicable, details of automation tools used in the process.	Page 3
Data items	10a	List and define all outcomes for which data were sought. Specify whether all results that were compatible with each outcome domain in each study were sought (e.g., for all measures, time points, analyses), and if not, the methods used to decide which results to collect.	Pages 3 and 4
	10b	List and define all other variables for which data were sought (e.g., participant and intervention characteristics, funding sources). Describe any assumptions made about any missing or unclear information.	Not applicable
Study risk-of-bias assessment	11	Specify the methods used to assess risk of bias in the included studies, including details of the tool(s) used, how many reviewers assessed each study and whether they worked independently, and, if applicable, details of automation tools used in the process.	Not applicable
Effect measures	12	Specify for each outcome the effect measure(s) (e.g., risk ratio, mean difference) used in the synthesis or presentation of results.	Not applicable
Synthesis methods	13a	Describe the processes used to decide which studies were eligible for each synthesis (e.g., tabulating the study intervention characteristics and comparing against the planned groups for each synthesis (item #5)).	Not applicable
	13b	Describe any methods required to prepare the data for presentation or synthesis, such as handling of missing summary statistics or data conversions.	Not applicable
	13c	Describe any methods used to tabulate or visually display results of individual studies and syntheses.	Pages 4 and 11
	13d	Describe any methods used to synthesize results and provide a rationale for the choice(s). If meta-analysis was performed, describe the model(s), method(s) to identify the presence and extent of statistical heterogeneity, and software package(s) used.	Not applicable
	13e	Describe any methods used to explore possible causes of heterogeneity among study results (e.g., subgroup analysis, meta-regression).	Not applicable
	13f	Describe any sensitivity analyses conducted to assess robustness of the synthesized results.	Not applicable
Reporting bias assessment page	14	Describe any methods used to assess risk of bias due to missing results in a synthesis (arising from reporting biases).	Not applicable
Certainty assessment	15	Describe any methods used to assess certainty (or confidence) in the body of evidence for an outcome.	Not applicable
**RESULTS**			
Study selection	16a	Describe the results of the search and selection process, from the number of records identified in the search to the number of studies included in the review, ideally using a flow diagram.	Page 12, Figure 1
	16b	Cite studies that might appear to meet the inclusion criteria, but which were excluded, and explain why they were excluded.	Not applicable
Study characteristics	17	Cite each included study and present its characteristics.	Table 1
Risk of bias in studies	18	Present assessments of risk of bias for each included study.	Not applicable
Results of individual studies	19	For all outcomes, present, for each study: (a) summary statistics for each group (where appropriate) and (b) an effect estimate and its precision (e.g., confidence/credible interval), ideally using structured tables or plots.	Not applicable
Results of syntheses	20a	For each synthesis, briefly summarize the characteristics and risk of bias among contributing studies.	Not applicable
	20b	Present results of all statistical syntheses conducted. If meta-analysis was done, present for each the summary estimate and its precision (e.g., confidence/credible interval) and measures of statistical heterogeneity. If comparing groups, describe the direction of the effect.	Not applicable
	20c	Present results of all investigations of possible causes of heterogeneity among study results.	Figure 2, Table 2
	20d	Present results of all sensitivity analyses conducted to assess the robustness of the synthesized results.	Not applicable
Reporting biases	21	Present assessments of risk of bias due to missing results (arising from reporting biases) for each synthesis assessed.	Not applicable
Certainty of evidence	22	Present assessments of certainty (or confidence) in the body of evidence for each outcome assessed.	Not applicable
**DISCUSSION**			
Discussion	23a	Provide a general interpretation of the results in the context of other evidence.	Page 16–19
	23b	Discuss any limitations of the evidence included in the review.	Page 18
	23c	Discuss any limitations of the review processes used.	Page 18
	23d	Discuss implications of the results for practice, policy, and future research.	Pages 18 and 19
**OTHER INFORMATION**			
Registration and protocol	24a	Provide registration information for the review, including register name and registration number, or state that the review was not registered.	Page 11
	24b	Indicate where the review protocol can be accessed, or state that a protocol was not prepared.	Page 11
	24c	Describe and explain any amendments to information provided at registration or in the protocol.	Not applicable
Support	25	Describe sources of financial or non-financial support for the review, and the role of the funders or sponsors in the review.	Page 20
Competing interests	26	Declare any competing interests of review authors.	Page 20
Availability of data, code and other materials	27	Report which of the following are publicly available and where they can be found: template data collection forms; data extracted from included studies; data used for all analyses; analytic code; any other materials used in the review.	Page 20

From: Page et al. (2021) [59].
